# Elucidating immunological characteristics of the adenoma-carcinoma sequence in colorectal cancer patients in South Korea using a bioinformatics approach

**DOI:** 10.1038/s41598-024-56078-2

**Published:** 2024-05-02

**Authors:** Jaeseung Song, Daeun Kim, Junghyun Jung, Eunyoung Choi, Yubin Lee, Yeonbin Jeong, Byungjo Lee, Sora Lee, Yujeong Shim, Youngtae Won, Hyeki Cho, Dong Kee Jang, Hyoun Woo Kang, Jong Wha J. Joo, Wonhee Jang

**Affiliations:** 1 Department of Life Sciences, Dongguk University, Seoul, 04620 South Korea; 2 Department of Internal Medicine, Dongguk University Ilsan Hospital, Dongguk University College of Medicine, Goyang, 10326 South Korea; 3 Division of AI Software Convergence, Dongguk University-Seoul, Seoul, 04620 South Korea; 4 Department of Internal Medicine, Seoul Metropolitan Government Seoul National University Boramae Medical Center, Seoul, 07061 South Korea; 5 Department of Computational Biomedicine, Cedars-Sinai Medical Center, Hollywood, CA USA

**Keywords:** Colorectal cancer, Advanced colorectal adenoma, Adenoma-carcinoma sequence, Immune repertoire, Cancer genomics, Cancer microenvironment, Gastrointestinal cancer, Computational biology and bioinformatics, Systems biology

## Abstract

Colorectal cancer (CRC) is one of the top five most common and life-threatening malignancies worldwide. Most CRC develops from advanced colorectal adenoma (ACA), a precancerous stage, through the adenoma-carcinoma sequence. However, its underlying mechanisms, including how the tumor microenvironment changes, remain elusive. Therefore, we conducted an integrative analysis comparing RNA-seq data collected from 40 ACA patients who visited Dongguk University Ilsan Hospital with normal adjacent colons and tumor samples from 18 CRC patients collected from a public database. Differential expression analysis identified 21 and 79 sequentially up- or down-regulated genes across the continuum, respectively. The functional centrality of the continuum genes was assessed through network analysis, identifying 11 up- and 13 down-regulated hub-genes. Subsequently, we validated the prognostic effects of hub-genes using the Kaplan–Meier survival analysis. To estimate the immunological transition of the adenoma-carcinoma sequence, single-cell deconvolution and immune repertoire analyses were conducted. Significant composition changes for innate immunity cells and decreased plasma B-cells with immunoglobulin diversity were observed, along with distinctive immunoglobulin recombination patterns. Taken together, we believe our findings suggest underlying transcriptional and immunological changes during the adenoma-carcinoma sequence, contributing to the further development of pre-diagnostic markers for CRC.

## Introduction

Colorectal cancer (CRC) is the third most common cancer type and the fourth leading cause of cancer deaths in 2020. The incidence of CRC has shown steady growth in South Korea for decades^1^. According to a recent study, CRC was the fourth most common cancer type and caused the third most cancer deaths in South Korea^1^. CRCs arise from their pre-malignant stage via the adenoma-carcinoma sequence and are affected by multiple genetic and/or environmental factors^2,3^. Given that, studying the genetic or molecular features of CRC precursors and CRCs may help explain the adenoma-carcinoma sequence and their early prevention.

One of the major classes of CRC precursors is advanced colorectal adenoma (ACA)^4^. Colorectal polyps whose diameter is greater than 1 cm are usually considered ACA. While the histological characteristics of ACA were mainly studied, transcriptomic-level information about pre-malignant polyps during the adenoma-carcinoma sequence remains insufficient. Previous studies indicated that genome-wide expression changes in transcripts such as *tumor protein D52-like 1*, *colon cancer-associated transcript 1*, or *urothelial cancer-associated 1* are involved with malignant transformation toward CRC^5,6^. Since previous approaches frequently lacked a size-dependent classification of ACA, there may still be unresolved features of ACA that need to be elucidated.

Along with the significant technical progress in single-cell genomics, the depletion or amplification of tumor-infiltrating immune cell-types regulates the tumor microenvironment (TME). Recently, Becker et al*.* conducted single-cell-level multi-omics analysis for the CRC continuum and identified distinctive exhausted and regulatory T-cell populations that progressed proportionally with malignancy^7^. Innate immune cells derived from myeloid progenitors such as macrophages, dendritic cells, and neutrophils have been associated with tumor progression or TME formation in CRC^8^. Molecular components comprising the immune repertoire, such as immunoglobulins (Igs), T-cell receptors (TCRs), and human leukocyte antigens (HLA), which are mainly secreted or presented by immune cells, have recently been highlighted for their association with the carcinogenesis and prognosis of CRC^9,10^. Thus, an integrative approach to delineating these immunological changes is indispensable for understanding the dynamic changes across the adenoma-carcinoma sequence.

**Figure 1:**
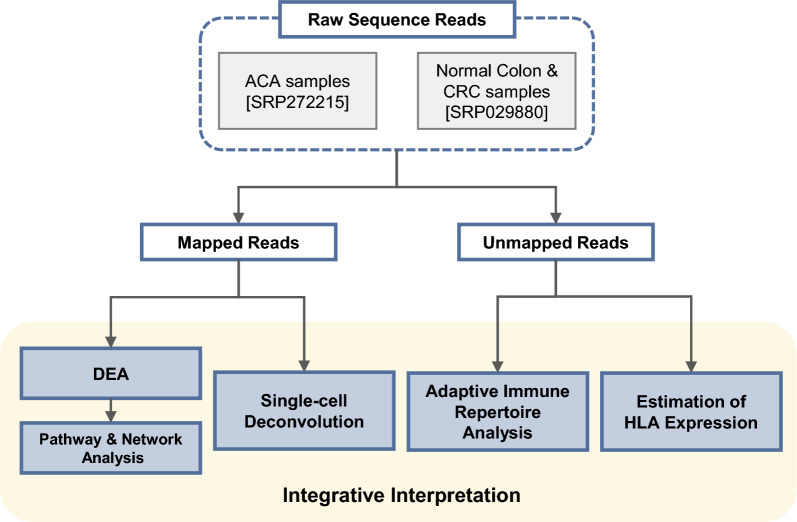
Schematic workflow of the overall analysis. To analyze continuous changes across adenoma-carcinoma sequences, ACA samples collected from Dongguk University Ilsan Hospital in Goyang, South Korea, were integratively analyzed with CRC and normal colon RNA-seq data obtained from South Koreans using the sequence read archive (SRA). After aligning raw sequences to the reference genome, sequences with corresponding genes were subsequently analyzed through DEA, pathway, network, and single-cell deconvolution analyses. Unmapped reads may contain hypermutated human genes such as *Ig*, *TCR*, and *HLA*, so we extracted reads matching these regions and conducted adaptive immune repertoire and HLA analyses.

Despite the importance of country- and ancestry-specific studies to ensure genetic and environmental consistency in identifying the transcriptional characteristics of pre-malignant polyps, only a few accessible data have been deposited in public databases. Particularly data derived from the Northeast Asian region and ancestry are extremely rare. Therefore, we generated RNA-sequencing (RNA-seq) data for 40 ACA samples and integrated it with publicly available RNA-seq data for CRC and normal adjacent colon tissues from 18 CRC patients with matching region and ancestry. We conducted differential expression analysis (DEA) across the colon stages (normal, ACA, and CRC) to identify the hub-genes that play a crucial role in CRC tumorigenesis. Parallelly, we analyzed the immunological features using single-cell deconvolution analysis with cell-type identification by estimating relative subsets of RNA transcripts X (CIBERSORT X) software and immune repertoire analysis by extracting the Ig, TCR, or HLA sequences from unmapped reads using the immune profiling by read origin protocol (ImReP) and seq2HLA software^11–13^. Overall process of the study is depicted at Fig. 1. Our approach may provide integrative transcriptional and immunological transitions across the CRC continuum, which may offer insights into the sequential carcinogenic mechanisms of CRC in the Northeast Asian or South Korean population with rare public availability.

## Results

### Identifying transcriptomic changes throughout the adenoma-carcinoma sequence

To investigate the ACA transcriptomic profiles and uncover the gene expression changes through the colorectal adenoma-carcinoma sequence, we performed and processed RNA-seq data obtained from 40 ACA patient samples and collected RNA-seq data for CRC and normal adjacent colon from a public database (SRA ID: SRP029880; CRC: 18; normal adjacent colon: 18). All ACA donors had polyps with diameters greater than 1 cm, and their histological types and the location from which the samples were obtained were recorded. Supplementary Table S1 provides ACA sample-specific information, while Supplementary Fig. S1 depicts colonoscopy images for each ACA subtype.

In order to reduce confounding factors, RNA-seq counts were normalized and batch effects between data sources were adjusted (Supplementary Fig. S2). Before conducting the DEA, potential outliers were screened with exploratory data analysis using multidimensional scale (MDS) plotting and principal component analysis (PCA). In both MDS and PCA plots, only one ACA sample (ACA_18) showed the extraordinary expression pattern, which was located in the opposite region without any other samples nearby. It was considered as an outlier and excluded from the subsequent analysis to obtain robust results (Supplementary Fig. S2).

**Figure 2:**
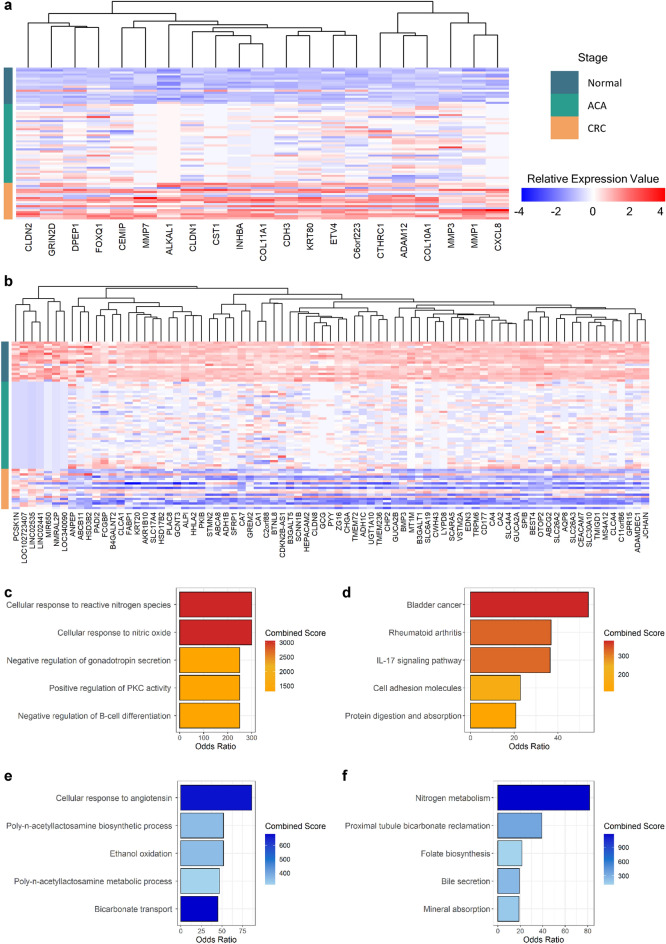
DEA for continuous DEG identification and their biological functions. (**a**) A heatmap showing up-regulated continuous DEG expression patterns across adenoma-carcinoma sequences. The annotation bar indicates sample type (dark green: normal, green: ACA, and orange: CRC). The color of each cell is proportionate to its column-centered relative expression value. (**b**) A heatmap of down-regulated continuous DEG expression levels. (**c**) A bar plot displaying the top 5 enrichments of up-regulated continuous DEGs using GO-BP pathways. The bar color indicates the combined score from enrichR, and the x-axis shows the OR of each pathway. (**d**) A bar plot of the enrichment test results for up-regulated continuous DEGs using KEGG pathways. (**e**) A bar plot showing the enrichment of down-regulated continuous DEGs on GO-BP pathways. (**f**) A bar plot of the enrichment of down-regulated continuous DEGs on KEGG pathways. The pathway enrichment analysis results are provided as Supplementary Data 4.

We conducted DEA to identify genes with continuous changes in expression value across the adenoma-carcinoma sequence (Supplementary Fig. S3 and Supplementary Data 1–3). Based on the fold-change (FC) between consecutive stages (Normal-ACA and ACA-CRC), there were 21 up-regulated (log_2_FC_ACA-Normal_ > 1, log_2_FC_CRC-ACA_ > 1, and false discovery rate (FDR) < 0.05) and 79 down-regulated genes (log_2_FC_ACA-Normal_ < − 1, log_2_FC_CRC-ACA_ < − 1, and FDR < 0.05), which were identified as continuous differentially expressed genes (DEGs). The full summary statistics from DEA for continuous DEGs are provided in Supplementary Tables S2 and S3. By applying the gene-wise scaling, we observed the gradual changes in gene expression levels on the continuum from normal tissue to CRC (Fig. 2a,b).

To identify the biological mechanisms of continuous DEGs, we conducted enrichment tests for gene ontology biological processes (GO-BP) and Kyoto Encyclopedia of Genes and Genomes (KEGG) pathways^14,15^. Up-regulated continuous DEGs were significantly enriched (*P* < 0.05) in pathways related to carcinogenesis, inflammation, and immune responses (Fig. 2c,d). Enrichment profiles of down-regulated continuous DEGs mainly implied the loss of normal colonic functions during carcinogenesis (Fig. 2e,f). Reflecting the carcinogenic role of reactive nitrogen species, related pathways showed the two highest scores and odds ratios (OR) in the enrichment analysis using up-regulated continuous DEGs in GO-BP categories, while down-regulated continuous DEGs showed the highest enrichment of nitrogen metabolism in the KEGG pathway (Fig. 2f). Significant enrichments of immune or inflammatory pathways such as B-cell differentiation, rheumatoid arthritis, and interleukin (IL)-17 pathways in Fig. 2c,d may suggest the importance of immunological landscapes in the adenoma-carcinoma sequence.

### Hub-genes for the adenoma-carcinoma sequence may also be important for patient survival

**Figure 3:**
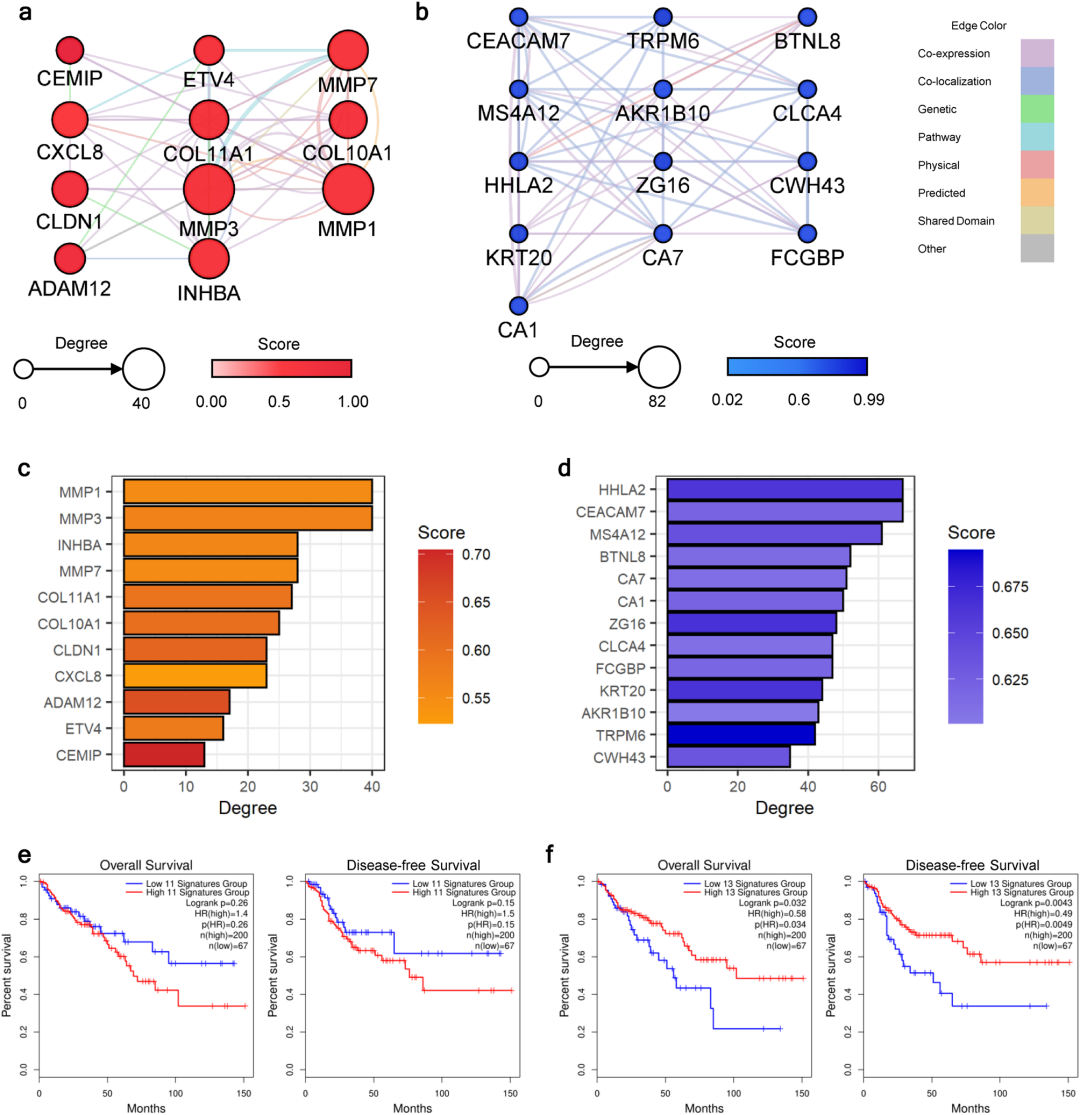
Identification of the carcinogenesis-stimulating hub-genes. PPI networks were constructed with (**a**) up- and (**b**) down-regulated hub-genes. The node sizes are proportional to their degrees. Darker node colors indicate higher scores calculated from the GeneMania plug-in, which delineates the functional importance of the gene. The edge colors depict interaction types between nodes. Bar plots depicting the network statistics of identified hub-genes for (**c**) up- and (**d**) down-regulated networks. The bar lengths indicate the degree of each gene, and the darker color indicates a higher score from the GeneMania plug-in. The survival analysis results using the (**e**) up- and (**f**) down-regulated hub-genes. Both the overall and disease-free survival rates were analyzed, and their results are presented on the left and right sides of each panel, respectively. For each input gene set, survival curves for patients with high or low expression levels are marked with red or blue lines, respectively.

To identify the functional or regulatory interconnection within the up- or down-regulated continuous DEGs, we constructed the protein–protein interaction (PPI) networks for the up- and down-regulated continuous DEGs individually. Interactions between each gene–gene pair were searched for their co-expression, co-localization, genetic relationship, involvement in the same pathway, physical interaction, computationally predicted interaction, shared domain, and others (Supplementary Fig. S4). After constructing the initial network, we computed the network statistics to identify the core-functioning or core-interacting genes among each network. We narrowed down the hub-genes by applying the cut-off of > 50% percentile for two criteria: degree and score from the GeneMania plug-in. Among 41 genes in the network of up-regulated continuous DEGs, 11 genes passed the cut-off value (Fig. 3a,c). Implying the transition into the malignant stage, genes related to cell migration, extracellular matrix (ECM) degradation, or rearrangement, such as *matrix metalloproteinase* (*MMP*), *collagen* (*COL*) family genes, *ADAM metallopeptidase domain 12* (*ADAM12*), and *cell migration-inducing hyaluronidase 1* (*CEMIP*), were identified as the network hub-genes. Additionally, *CXC motif chemokine ligand 8* (*CXCL8*) was included in the criteria, which is known for its critical role in tumor microenvironment (TME) alteration^16^. In a down-regulated network, 13 genes were identified as hub-genes (Fig. 3b,d). Genes related to the carcinogenesis of the gastrointestinal tract, such as *carcinoembryonic antigen cell adhesion molecule 7* (*CEACAM7*), *membrane-spanning 4-domains A12* (*MS4A12*), *carbonic anhydrase* (*CA*) *1* and *7*, *chloride channel accessory 4* (*CLCA4*), *Fc gamma binding protein* (*FCGBP*), and *aldo–keto reductase family 1 member B10* (*AKR1B10*), were involved in the list of hub-genes. Other genes identified were related to biological functions in immunological regulation (*HERV-H LTR-associating 2*: *HHLA2*, and *zymogen granule protein 16*: *ZG16*) and cellular structure formation (*keratin 20*: *KRT20*, and *cell wall biogenesis 43 C-terminal homolog*). Conforming with the previous studies, this results collectively implied that the overactivated ECM remodeling and malfunctioning immune cells are the crucial component in the progression toward the CRC stage^17,18^.

In order to validate the population-specificity and generalizability of the hub-genes, we compared the direction of gene expression changes against external datasets. We collected one representative dataset for each population: Northeast Asian (NEA), European (EUR), and mixed (multi-study meta-analysis) (Table 1)^5,6,19^. By calculating the Pearson’s correlation coefficients (PCC) of the log2FC values between our results and external studies, we observed positive correlations except for the CRC-ACA difference in EUR dataset (Supplementary Table S4) and our results showed high similarity with the DEA results in the NEA dataset (0.522 < PCC < 0.924). Since there were only few overlapping genes with multi-study meta-analysis (6, 6, and 2 genes for ACA-normal, CRC-normal, and CRC-ACA, respectively), we found that all of the directions of differential expression were identical. These results may imply the underlying population-specific mechanisms in ACA to CRC progression; however, the difference in gene expression measurement platform in EUR dataset (microarray) in itself may have caused the technical variation.

**Table 1:** The list of the external datasets used for validation.

PMID	Population	Normal	Adenoma	CRC	Method
34458146	NEA (China)	5	5	5	RNA-seq
31694571	EUR (Hungary)	20	20	20	Microarray
34944753	Mixed (meta-analysis)	105	155	205	Microarray

Differential expression statistics from original publication (PMID 34458146 and PMID 34944753) or GEO2R (PMID 31694571) were used for comparison.

Subsequently, we validated the importance of the hub-genes by examining the association between gene expression levels and patient survival. In the survival analysis, we examined the overall and disease-free survival rates using colorectal adenocarcinoma (COAD) data from The Cancer Genome Atlas (TCGA). As shown in Fig. 3e, patients with high expression levels of up-regulated hub-genes showed a lower survival rate with nominal statistical significance (*P* overall survival = 0.26 and *P* disease-free survival = 0.15). Survival rates significantly dropped (*P* < 0.05) in both overall and disease-free survival when the gene expression levels of down-regulated hub-genes were in the lower 25% range (Fig. 3f). Together, we found that the hub-genes from our analyses may crucially affect all stages of disease prognosis, from disease progression to relapse to the survival rate of patients.

### Continuous changes were estimated in the landscape of innate immunity cells

Considering the emerging role of immune cell composition in TME development, we conducted single-cell deconvolution analysis to estimate the immune cell type fraction across the adenoma-carcinoma sequence. Examining the immune cell types involved in innate immunity, there were three cell types that showed a certain trend from a normal state to CRC.

**Figure 4:**
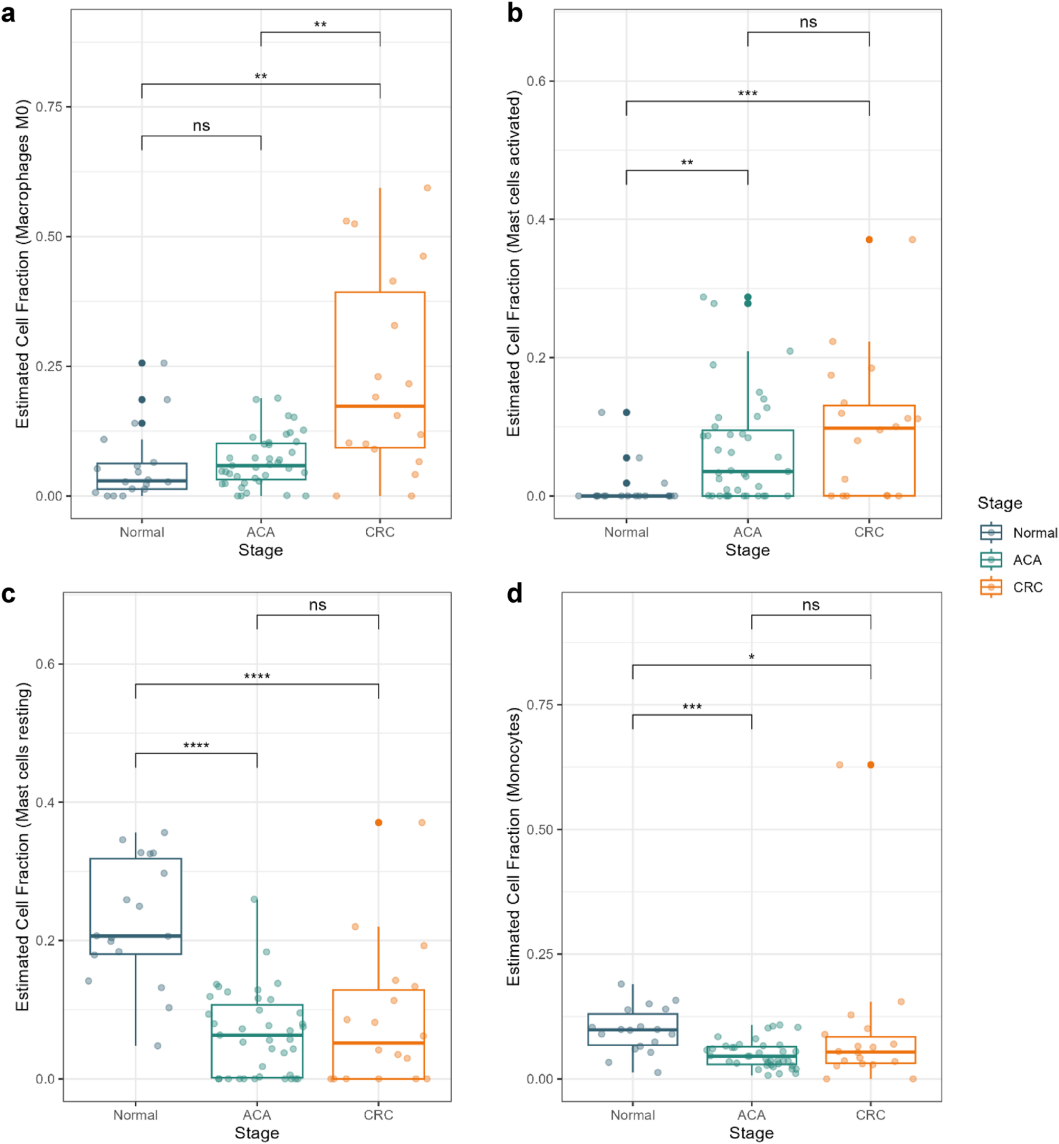
Cell type fraction estimation for innate immunity cells. Box plots presenting the estimated cell fractions calculated with CIBERSORT X software. Cell fractions for (**a**) macrophage M0, (**b**) activated mast cells, (**c**) resting mast cells, and (**d**) monocytes are presented. Normal, ACA, and CRC sample types are marked in blue, green, and yellow colors, respectively. **** *P* < 0.0001, *** *P* < 0.001, ** *P* < 0.01, * *P* < 0.05, and ns > 0.05.

Consistent with previous findings suggesting the recruitment of M0 macrophages in TME, our results showed a significantly high cell fraction of M0 macrophages in CRC samples (Fig. 4a)^20^. Recruited macrophages seem to be actively polarized into both M1 and M2 states in CRC, which distinguishes it from ACA and normal tissues (Supplementary Fig. S5). Macrophages are known for their role in TME regulation by secreting cytokines that promote tumor cell proliferation. Our results demonstrate that this critical transition of macrophage proportion does not appear at the pre-malignant stage, but a rapid shift can occur when the major proportion of the tissue turns malignant.

Mast cells were another form of innate immune cell that demonstrated a significant difference. Based on a previous study by Yu et al*.*, cross-talk between tumor-resident mast cells and surrounding cancer cells promotes tumor growth by releasing protumorigenic signals^21^. Our results suggest that in ACA and CRC tissues, tumor-resident mast cells were more likely to be in an active state, whereas mast cells in normal tissue were mostly in a resting state (Fig. 4b,c). Additionally, a significant depletion of the monocyte population was estimated in ACA and CRC samples (Fig. 4d). While previous studies had mainly discussed the negative correlation between monocyte abundance and CRC prognosis, we suggest that this change might start from premalignant state of CRC^22^.

We then constructed the multinomial logistic regression models for classifying the tissue stage using the estimated cell fractions for macrophages and monocytes (see Methods). By examining the accuracy of the constructed models, we found that these predictors can well classify the tissue stages up to 88% of accuracy (Supplementary Figure S6). We also tested the regression models with single cell type. While macrophage showed its potential to be a useful predictor (70% < accuracy), monocyte alone showed the poor property as the prognostic marker (accuracy = 56%). This results may imply that the cell fraction of macrophage can serve as the prominent prognostic/diagnostic marker for CRC continuum.

### Composition of adaptive immune cells and immune repertoires are associated with CRC development

**Figure 5:**
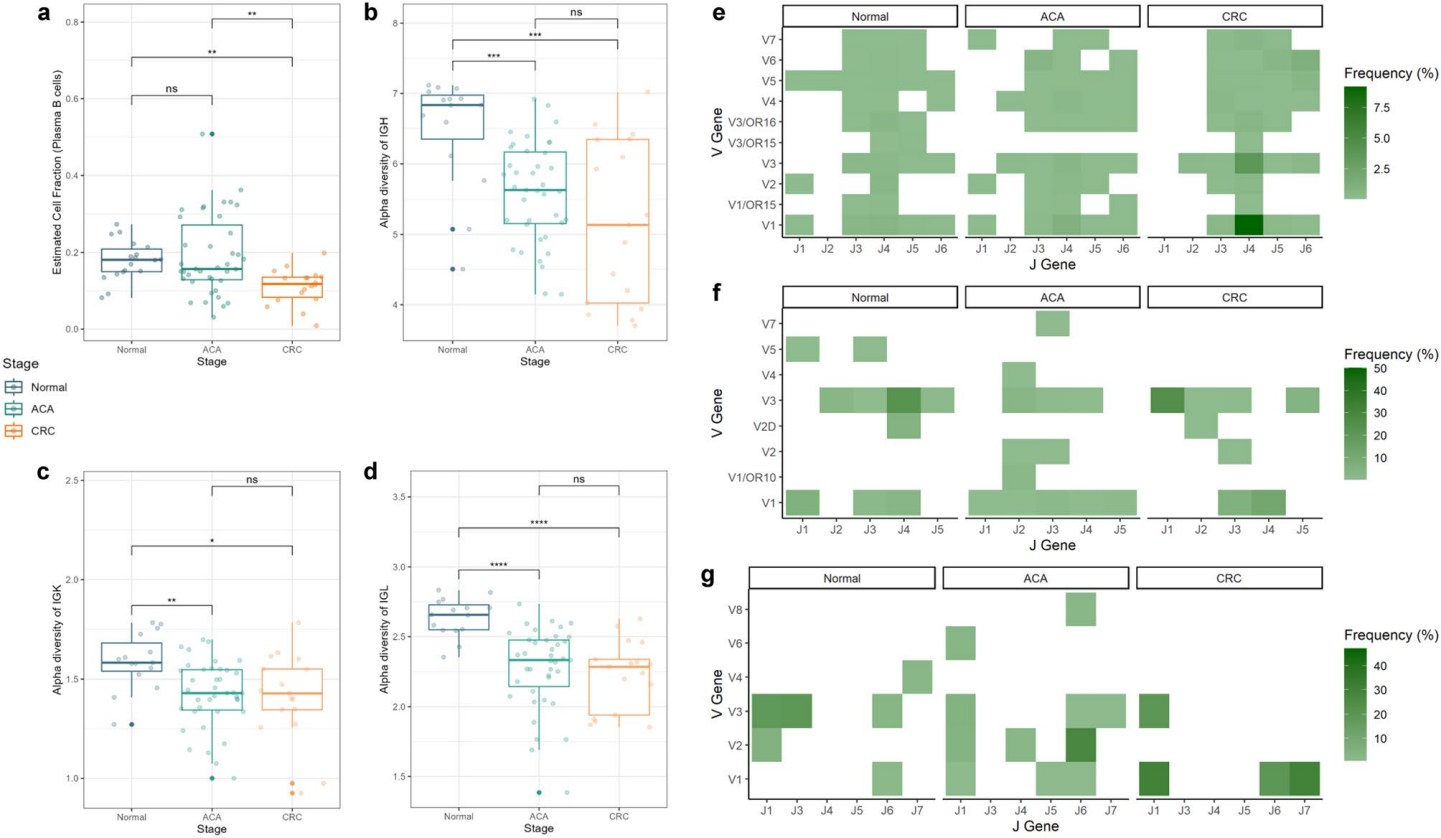
Cell fraction and immune repertoire analysis for adaptive immunity components across the CRC continuum. (**a**) Box plots presenting the plasma B-cell fraction across the CRC continuum. The graph colors indicate the sample types (normal: blue; ACA: green; CRC: yellow). The box plots of the immune repertoire diversity analysis are: (**b**) IGH, (**c**) IGK, and (**d**) IGL chains. The heatmaps delineate the VJ recombination frequencies of (**e**) IGH, (**f**) IGK, and (**g**) IGL chains across the adenoma-carcinoma sequences. The color of each cell is proportional to the relative frequency of VJ recombination in certain sample types.

Tumor-infiltrating adaptive immune cells, including B- and T-cells, are involved in TME formation and immune evasion mechanisms^23^. Single-cell deconvolution analysis showed a significant depletion of the plasma B-cell population, which is a B-cell subtype that mostly secretes Igs (Fig. 5a). Additionally, we found increased cell abundance in memory B-cells in ACA samples and a nominally decreasing trend in naive B-cells in ACA and CRC samples (Supplementary Fig. S7).

To analyze the heterogeneity of Ig sequences among the samples, we extracted the unmapped reads matching Ig sequences using the ImReP software. In this procedure, four out of 40 samples (SRR975551, SRR975573, SRR975574, and SRR975577) exhibited ambiguous Ig sequences, which can be considered poor-quality reads and were excluded from subsequent analysis. We calculated the alpha diversity index, which describes sequence diversity within the samples, to evaluate the heterogeneity of Ig repertoires. While the decrease in plasma cell population does not become obvious in CRC samples, we found that the Ig repertoire started to decrease from the premalignant stage of CRC. This trend was observed for complementarity-determining region 3 (CDR3) sequences of all three types of Ig chains: IgH, IgK, and IgL (Fig. 5b–d).

Along with the significant decrease in Ig diversity, we could identify the typical recombination patterns of the V and J segments of Igs (Fig. 5e–g). Specifically, the combination of V3-J2 and V6-J6 segments in IgH, V2-J3 segments in IgK, and V1-J1 segments in the IgL chain were only observed in samples from ACA and CRC stages, suggesting a potential association between the VJ recombination patterns and CRC progression. Analyzing the diversity of Ig repertoires between the samples with beta diversity calculation, we found that the IgH chains between samples from normal stages were relatively similar (Sørensen-Dice index: 0.409; Supplementary Fig. S8). We also identified a significant decrease in the CD8 T-cell population; however, we were unable to detect conforming patterns for TCR diversity (Supplementary Figs. S9–S11) because we were unable to capture sufficient sequences corresponding to the T-cell receptor (TCR) region in normal or CRC samples.

### Estimating tumor-associated HLA allele typing and expression analysis

While Igs or TCRs generally counteract the non-self antigens previously encountered by the host, HLA genes are also involved in self-antigen recognition, whose malfunctioning is closely related to the immune evasion of cancer cells^24^. Thus, we extracted the reads aligned to the HLA alleles with seq2HLA software^25^. To assess the mutation occurring through carcinogenesis, we profiled the HLA types across the patients. We found the changes between normal adjacent and CRC tissues for HLA-A (02:01) and HLA-C (07:02) among class I alleles, and DPA1 (01:03, 02:01, 02:03, and 03:02), DPB1 (02:01, 04:01, 04:02, 05:01, and 104:01), DQA1 (01:01 and 01:02), DQB1 (03:01, 03:03, 04:01, 05:01, 05:03, and 06:02), and DRB1 (13:02) on class II alleles (Supplementary Fig. S12). Considering that the normal adjacent tissue and CRC tissue were collected from paired patients, these changes can imply the somatic mutation of the HLA allele during the carcinogenesis.

**Figure 6:**
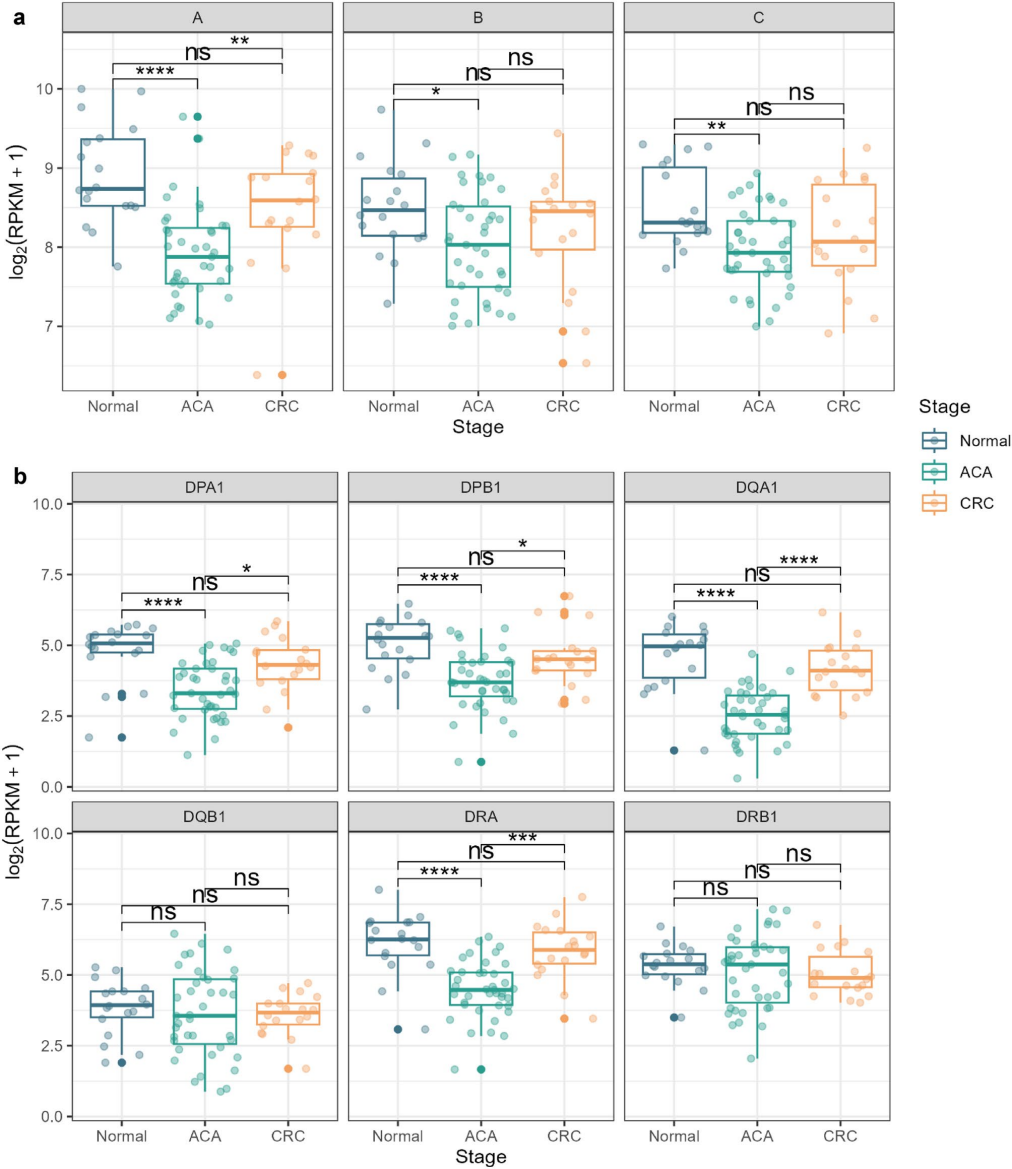
HLA gene expression analysis across the CRC continuum. (**a**) Box plots presenting the expression levels of type I HLA genes (HLA-A, HLA-B, and HLA-C) across adenoma-carcinoma sequences. (**b**) Box plots of the expression level of type II HLA genes (HLA-DPA1, HLA-DPB1, HLA-DQA1, HLA-DQB1, HLA-DRA, and HLA-DRB1). The graph color indicates the sample stage (normal: blue; ACA: green; CRC: yellow).

Subsequently, we analyzed the expression of HLA genes across samples using a non-parametric Kruskal–Wallis test, followed by Dunn’s test as a post-hoc test. We detected significantly low HLA class I gene expression levels in ACA samples (Fig. 6a). This trend was consistent in all three HLA class I alleles: HLA-A, HLA-B, and HLA-C. Even though the difference between normal and CRC tissue was not statistically significant, we note that there is a definite trend toward decreased CRC expression levels compared with normal tissue, with Z-scores of − 1.30 (*P* = 0.19), − 1.06 (*P* = 0.28), and − 1.75 (*P* = 0.07) for HLA-A, B, and C alleles, respectively. Significantly decreased expressions of HLA genes in ACA samples were similarly observed for HLA class II genes, except for HLA-DQB1 and DRB1 alleles (Fig. 6b). Along with HLA class I genes, the median expression level of HLA class II genes was highest in normal tissue. Based on these results, we suspect that immune evasion mechanisms mediated by HLA gene expression may begin at the ACA stage and be associated with somatic mutations at HLA loci.

## Discussion

Given that CRC arises via the adenoma-carcinoma sequence, a systematic study of the continuum from normal colon to CRC is necessary to elucidate the underlying nature of CRC pathogenesis. Confounding factors that may influence the gastrointestinal condition, such as eating habits, living environments, and genetic backgrounds, need to be constrained for the identification of such group-specific mechanisms of CRC^26,27^. Considering this characteristic of the CRC continuum, we narrowed our scope to South Korea, where the majority of the population is of NEA ancestry and the eating patterns do not deviate significantly from typical South Korean^28^. Previous observational study by Park et al*.*, suggested that Westernized eating habits are correlated with the CRC risk, which are rapidly increasing in South Korea^29^. Combinatorial effects between NEA ancestry with Westernized eating habits may deduce characteristic effects in molecular level. Transcriptomic signatures exhibited a continuous transcriptional imbalance proportional to the clinical stage; consequently, their biological functions were closely related to the carcinogenic processes (Fig. 2). Other than terms explicitly implicating the loss of intestinal digestive functions or cancerous changes, our functional annotation results highlighted nitrogen metabolism and immune-related inflammatory pathways (Fig. 2c–f). Previous studies have shown that CRC patients have a high concentration of reactive nitrogen species (RNS), which can alter the TME landscape by stimulating inflammatory cascades^30–32^. Our results support these previous findings and additionally suggest that RNS-induced inflammation may play a crucial role in the premalignant stage of the CRC continuum.

We confirmed that the majority of the hub-genes identified across the adenoma-carcinoma sequence have promising associations with CRC at the clinical level. Among them, two up-regulated (*ADAM12*, *CEMIP*) and one down-regulated (*HHLA2*) hub-genes have not been majorly highlighted for their contribution to adenoma-carcinoma transition, to the best of our knowledge. These genes can be suggested as a novel prediagnostic marker in the ACA stage that can simultaneously predict the prognosis in CRC. A member of the metalloproteinase family, *ADAM12*, is known for its oncogenic role in CRC by regulating TME by ECM degradation and processing various chemokines and cytokines that can activate immune cell infiltration.^33^ Reflecting these activities, the gene expression level of *ADAM12* is strongly associated with poor patient survival and prognosis in various types of cancer, including colorectal, thyroid, lung, and pancreatic cancers^33–36^. *CEMIP* is a metastasis-related gene that is associated with tumor invasion, cell death, and metabolism reprogramming; however, its putative ability as a prediagnostic genetic marker was not discussed before^37–39^. A population-based study by Zhang et al*.* suggested the gene expression of *HHLA2* as the prognostic marker for four types of solid tumors (renal, intrahepatic, gastric, and lung cancers).^40^ Our results elucidated the substantial oncogenic role of *HHLA2* not only for the premalignant stage of CRC but also for CRC prognosis. Based on the role of HHLA2, whose gene product mainly localize to monocyte surface, regulates immune response and interrupt cell-mediated immunity by regulating T-cell proliferation, we propose this gene can be a potential link between the gene expression and immune cell population alteration in CRC.

Our results also supported the previous findings implicating the role of myeloid cells as the regulator of malignant TME formation, which is also associated with CRC prognosis^7,41,42^. Notably, while intensive recruitment of M0 macrophages followed by ambivalent polarization toward M1 or M2 states is known as the characteristic TME of CRC, relatively low (~ 20%) cell proportions for any state of macrophage were observed in samples at the ACA stage (Fig. 4a and Supplementary Fig. S5). Mast cells in the activated state were also increased through the adenoma-carcinoma sequence (Fig. 4b). Histamine secreted by activated mast cells can activate the carcinogenic beta-catenin pathway, which can accelerate adenoma formation^43,44^. Based on our deconvolution analysis, the monocyte population decreased during the adenoma-carcinoma sequence; however, considering that monocytes can play a bilateral role depending on the presence or absence of C–C chemokine receptor type 2 protein, this finding needs validation by deeper functional studies^45^. Additionally, considering that high Ig clonality and/or high expression levels of B-cell signature genes are correlated with better prognosis in various cancer types including, melanoma, lung cancer, pancreatic cancer, and head and neck squamous cell carcinoma, our results of a sequentially decreasing proportion of plasma B-cells and Ig diversities can suggest that this trend is also applicable to CRC^46^. In line with the Ig-specific deep profiling conducted by Zhang et al*.*, we sought to determine whether Ig sequences from samples in the CRC stage exhibited a relatively homogeneous Ig repertoire, which could be driven by Ig specification toward tumor-specific antigen^47^. Furthermore, we suggested that this specific Ig recombination pattern may arise from the recruitment of tumor-infiltrating B-cells at the premalignant stage (Fig. 5e–g).

In line with a previous study by Castro et al*.,* we found altered HLA allele types in CRC tissues, which may have occurred through somatic mutations during carcinogenesis^48^. Especially for somatic mutations in HLA class I genes, this change can cause the misrecognition of tumor neoantigens as self-antigens, which may interfere with the antigen-recognition mechanism and help tumor cells escape from immune system attacks. While there are several studies reporting the association between decreased expression of HLA class I or II genes and the metastatic potential of CRC by assisting the immune evasion mechanisms, our results exhibited a partially conforming pattern (Fig. 6)^49–51^. Additionally, for all HLA types, samples in the ACA stage showed the lowest expression level. We speculate that this characteristic expression pattern may cloak the ACA from the surrounding immune cells before the complete TME is formed or that this observed phenomenon may be due to the limitations of our study.

Herein, we performed an integrative study to delineate the characteristic changes during the adenoma-carcinoma sequence of CRC, mainly focusing on immunological changes. We combined general transcriptomic analysis with single-cell deconvolution and immune repertoire analyses, followed by HLA typing. Throughout these steps, we identified three novel genes associated with the adenoma-carcinoma sequence and immunological changes containing broad landscape alterations in innate immunity cells and a decreased plasma cell population with Ig diversity. Additionally, we mapped HLA allele types across the adenoma-carcinoma sequence and measured their gene expression levels. Because ACAs are surgically removed immediately once observed, it is difficult to observe transcriptome changes in a single polyp as it transforms into an ACA in a longitudinal study. We complemented this issue by utilizing the transcriptome data from normal adjacent colon and CRC with batch effect correction procedures, still, this study has numerous limitations. As previously stated, this was not a longitudinal study. Even normal tissues were not obtained from healthy subjects but from the adjacent normal colon of CRC patients. In addition, because our results are based on bulk tissue RNA-seq experiments, our findings need additional validation through single-cell or molecule-specific profiling methods. Despite these limitations, we verified that the majority of patterns we observed well conformed with the previously known ACA-CRC continuum characteristics. Moreover, we have shown the potential prognostic/diagnostic role of the hub-genes and cell fraction changes by survival analysis and multinomial regression model construction. Overall, we believe that our study identifies the key changes in the transcriptome and immunological landscape of the adenoma-carcinoma sequence in CRC. To the best of our knowledge, this is the first transcriptome-wide level study for CRC adenoma-carcinoma sequence in South Korean patients of predominantly NEA ancestry, focusing on immunological features of the CRC continuum.

## Methods

### Sample collection and preparation for RNA-seq

ACA samples were collected from 40 South Korean patients who visited Dongguk University Ilsan International Hospital (Goyang, South Korea). Dissected samples were immediately treated with Trizol (Sigma-Aldrich, St. Louis, MO, USA), and then the samples were stored at -80℃. RNA-seq was performed with Illumina NovaSeq 6000 in Macrogen (Seoul, South Korea) with a paired-end design.

### Data collection for patients with CRC and normal adjacent colon tissue

Considering the pathogenic nature of CRC, which can be highly country-specific, RNA-seq data from South Korean patients with CRC and normal adjacent colons were searched in SRA. Raw RNA-seq data for normal adjacent colon and CRC tissues were retrieved from 18 South Korean CRC patients from the public database in FASTQ format (SRA ID: SRP029880)^52^.

### Preprocessing of RNA-seq reads

RNA-seq raw data were retrieved, and adapter trimming was conducted with BBMap (ver. 38.81)^53^. Processed reads were then aligned to the reference genome (GRCh38.p13) from GENCODE (https://www.gencodegenes.org/) using the STAR aligner (ver. 2.7.3a, https://github.com/alexdobin/STAR) and saved in BAM-formatted files, while unmapped reads were saved within^54,55^. The BAM files were converted into read count data by SAMtools (ver. 1.10) and HTSeq (ver. 0.11.4)^56,57^.

### Preprocessing of count data and DEA

Raw read counts of RNA-seq data were normalized separately by the data sources (collected in this study or from a public database) with a variance stabilizing transformation method in the DESeq2 R package^58^. To merge the normalized datasets from different sources by correcting batch effects, inter-study variation was derived using the ComBat function in sva R package^59^. Additionally, the continuous DEGs between clinical stages were obtained using limma R package^60^.

### Downstream analysis of continuous DEGs

To investigate the biological functions of continuous DEGs, pathway-level enrichment was analyzed for GO and KEGG pathways with enrichR^14,15,61^. The interactions within up- or down-regulated continuous DEGs were defined using the GeneMania plug-in and visualized using Cytoscape (ver. 3.8.2.)^62,63^. Overall survival and disease-free survival analyses were conducted using the GEPIA2 web server (http://gepia2.cancer-pku.cn/) on the TCGA-COAD dataset^64,65^. A custom cut-off value of 25% was used to classify the high expression group (patients with an upper 25% expression value of input signatures) and the low expression group (patients with a lower 25% expression value).

### Validation of hub-genes by comparing with external datasets

To validate the robustness and check population-specific effects of hub-genes, we compared our results with the external dataset for NEA, EUR, and mixed-population datasets. The collected datasets were listed in Table 1. The results for PMID34458146 and PMID34944753 were retrieved from their original publications and the results for PMID31694571 were calculated by GEO2R. The similarity between our results and external datasets were compared by calculating PCC.

### Single-cell deconvolution analysis for merged dataset

The immune cell-type composition per sample was estimated using CIBERSORT X (https://cibersortx.stanford.edu/) in absolute output mode with automatic batch correction enabled^12^. Raw count values were converted into counts per million mapped reads with library size normalization as the input bulk gene expression matrix. To ensure accuracy, up to 500 permutations were analyzed.

### Construction of multinomial logistic regression models

To examine the potential properties of estimated macrophage and monocyte cell fractions, we constructed multinomial logistic regression models with nnet R package. We tested for 5 different combinations of cell fractions as predictor variables as below.

$$\mathrm{Model }1:{\text{ log}}\left(\frac{{p}_{j}}{{p}_{J}}\right)= {\beta }_{0}+ {\beta }_{1}M0+ {\beta }_{2}M1+ {\beta }_{3}M2+ {\beta }_{4}Mono$$

$$\mathrm{Model }2:{\text{ log}}\left(\frac{{p}_{j}}{{p}_{J}}\right)= {\beta }_{0}+ {\beta }_{1}M0+ {\beta }_{2}M2/M1+ {\beta }_{4}Mono$$

$$\mathrm{Model }1:{\text{ log}}\left(\frac{{p}_{j}}{{p}_{J}}\right)= {\beta }_{0}+ {\beta }_{1}M0+ {\beta }_{2}M1+ {\beta }_{3}M2$$

$$\mathrm{Model }1:{\text{ log}}\left(\frac{{p}_{j}}{{p}_{J}}\right)= {\beta }_{0}+ {\beta }_{1}Mono$$

$$\mathrm{Model }1:{\text{ log}}\left(\frac{{p}_{j}}{{p}_{J}}\right)= {\beta }_{0}+ {\beta }_{1}M1+ {\beta }_{2}M2+ {\beta }_{3}Mono$$

where *M0*, *M1*, and *M2* correspond to each state of macrophage fraction and *Mono* corresponds to the monocyte. The accuracy of each model was calculated as the percentage of correctly predicted label.

### Profiling of immune repertoires with unmapped RNA-seq reads

The reads unmapped onto the human reference genome in the alignment step of STAR software were used for this analysis following a read origin protocol^66^. The reads mapped onto the CDR3 sequences in Igs and TCR loci were analyzed per sample using ImReP (version 0.3, https://github.com/mandricigor/imrep)^13^. ImReP defines clones having identical CDR3 amino-acid sequences as clonotypes and identifies the corresponding V(D)J recombination. Two indexes were used to assess the heterogeneity of adaptive immune repertoires: alpha diversity (Shannon entropy) and beta diversity (Sørensen–Dice index). Briefly, the former indicates clonotypic diversity within the immune repertoire of a sample, while the latter indicates the compositional diversity of immune repertoires between samples. The alpha diversity of Igs and TCRs were respectively compared between clinical stages. The beta diversity of Igs and TCRs were respectively compared by all the combinations of clinical phenotype group pairs (e.g., normal *vs.* normal, normal vs. ACA).

### Estimation of HLA gene expression

To study the contribution of HLA regions to the immunological features of ACAs, the reads mapped onto the HLA reference sequences at chromosome 6 were analyzed by seq2HLA software (ver. 2.3)^25^. The HLA reads were then grouped by 18 loci (class I: 3, class II: 6, and class III: 9), and the locus-specific HLA gene expression levels were estimated by RPKM values. Due to the skewed distribution of gene expression levels, log-transformation as log_2_(RPKM + 1) was applied before conducting statistical analysis.

### Statistical analysis

Due to data skewness, a non-parametric Kruskal–Wallis test was conducted for group-by-group comparisons, followed by Dunn’s multiple comparison test as the post-hoc test with a *P*-value threshold of 0.05. All tests were performed using R (ver. 4.0.2), and the results were visualized with the R packages—pheatmap, ggplot2, ggfortify, and ggpubr.

### Ethical approval

Sample collection and study procedures were carried out in accordance with the Declaration of Helsinki. Any related ethical issues were approved by the institutional review board of Dongguk University Ilsan International Hospital (IRB No. 2019-02-009). Every patient and/or their legal guardians signed informed consent after receiving oral and written information.

### Supplementary Information

Supplementary Information 1.

Supplementary Information 2.

Supplementary Information 3.

Supplementary Information 4.

Supplementary Figures.

Supplementary Tables.

## Data Availability

RNA-seq data from ACA patients are deposited in NCBI-BioProject and GEO with the accession ID PRJNA646641 (raw fastq data) and GSE154548 (processed data), respectively. Raw RNA-seq data for paired CRC and adjacent normal stages are available in NCBI-SRA with the accession ID SRP029880.
