# Supplementary material for: Elucidating immunological characteristics of the adenoma-carcinoma sequence in colorectal cancer patients in South Korea using a bioinformatics approach
Source: Sci Rep. 2024 May 2;14:10105. doi: 10.1038/s41598-024-56078-2 (PMC11066069; doi:10.1038/s41598-024-56078-2)
Supplement: Supplementary file 5 — Supplementary Figures. [file 41598_2024_56078_MOESM5_ESM.pdf]

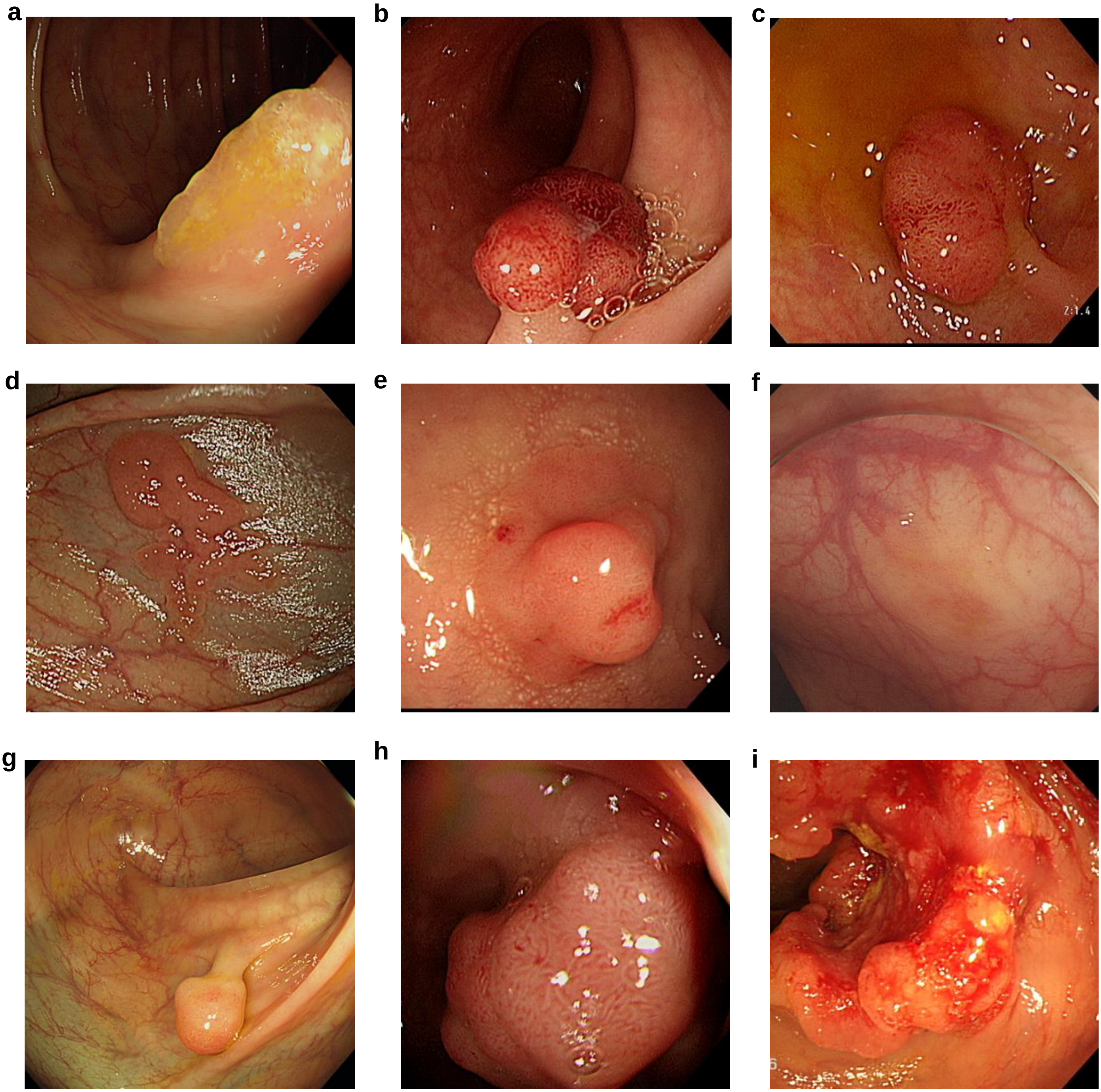

Supplementary Figure S1. Representative picture of each ACA subtypes taken by colonoscopy. Colonoscopy of (a) SSA/P, (b) TA with low grade dysplasia, (c) TA with high grade dysplasia, (d) TVA with low grade dysplasia, (e) TVA presenting high grade dysplasia, (f) HP, (g) IP, (h) IAC, and (i) AC.

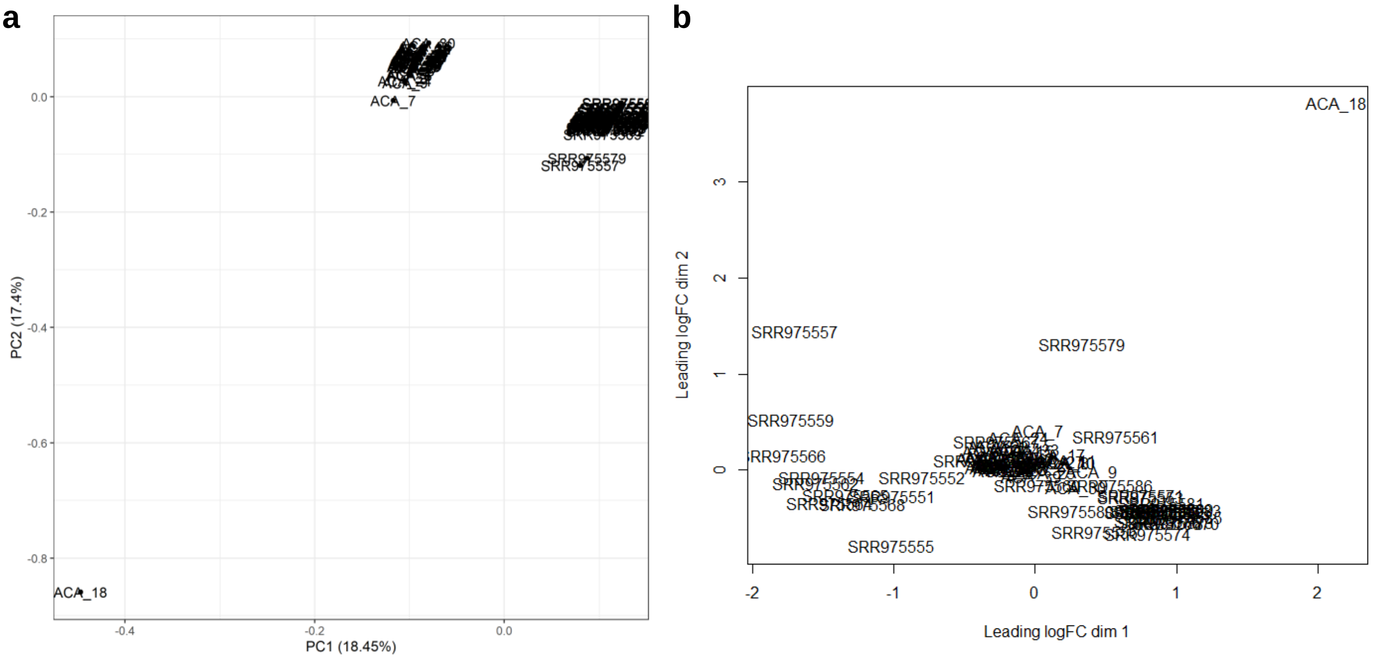

Supplementary Figure S2. Explanatory analysis with dimensional reduction. Dimensional reduction result from a) PCA and b) MDS.

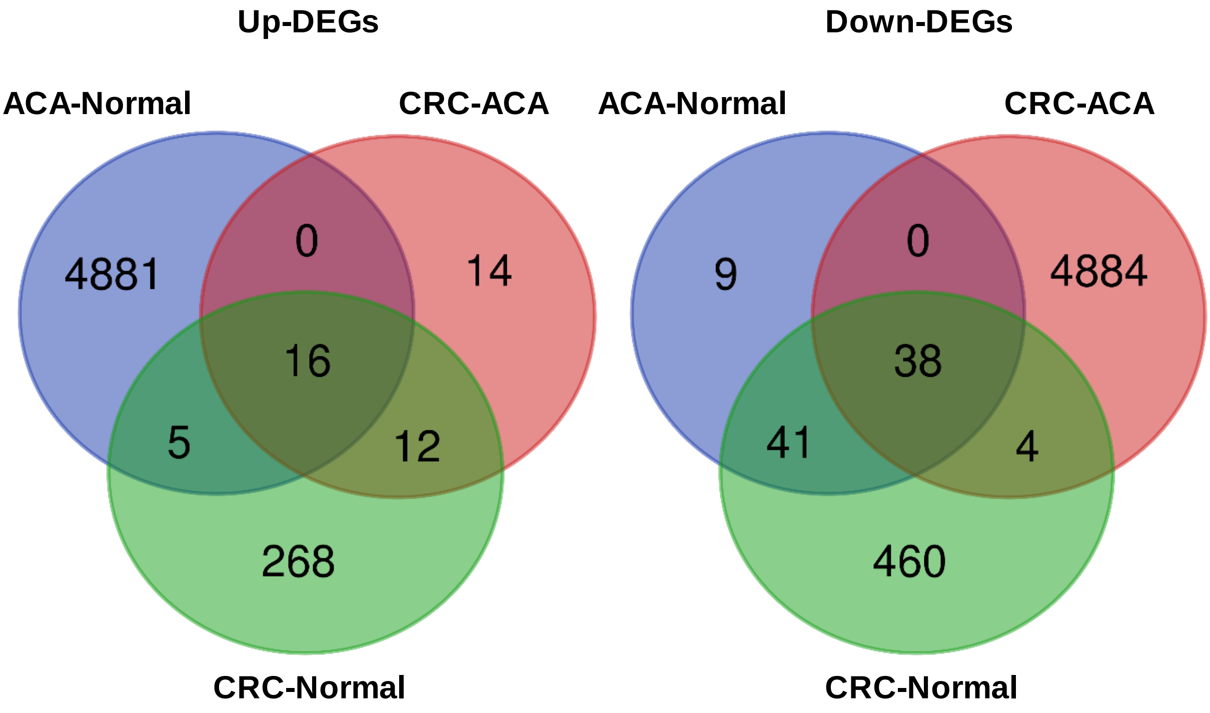

Supplementary Figure S3. Venn-diagrams depicting the overlaps between each stage.

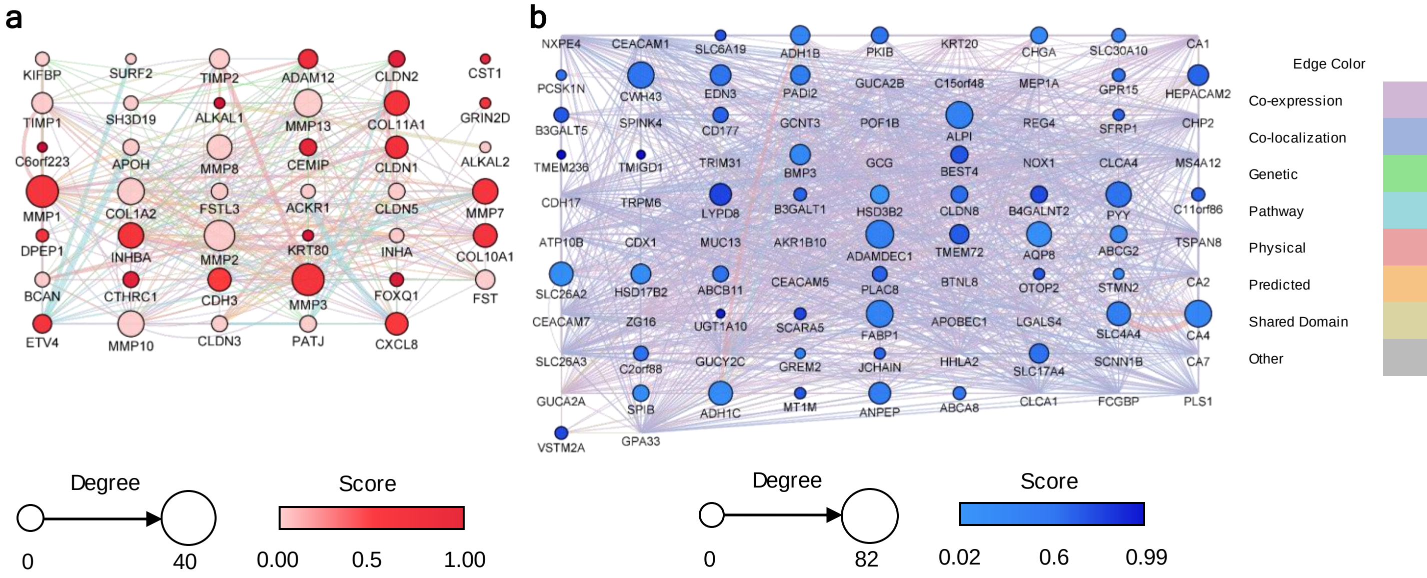

Supplementary Figure S4. Total network constructed with continuous DEGs

The network plots depicting the networks constructed with a) up- and b) down-regulated continuous DEGs.

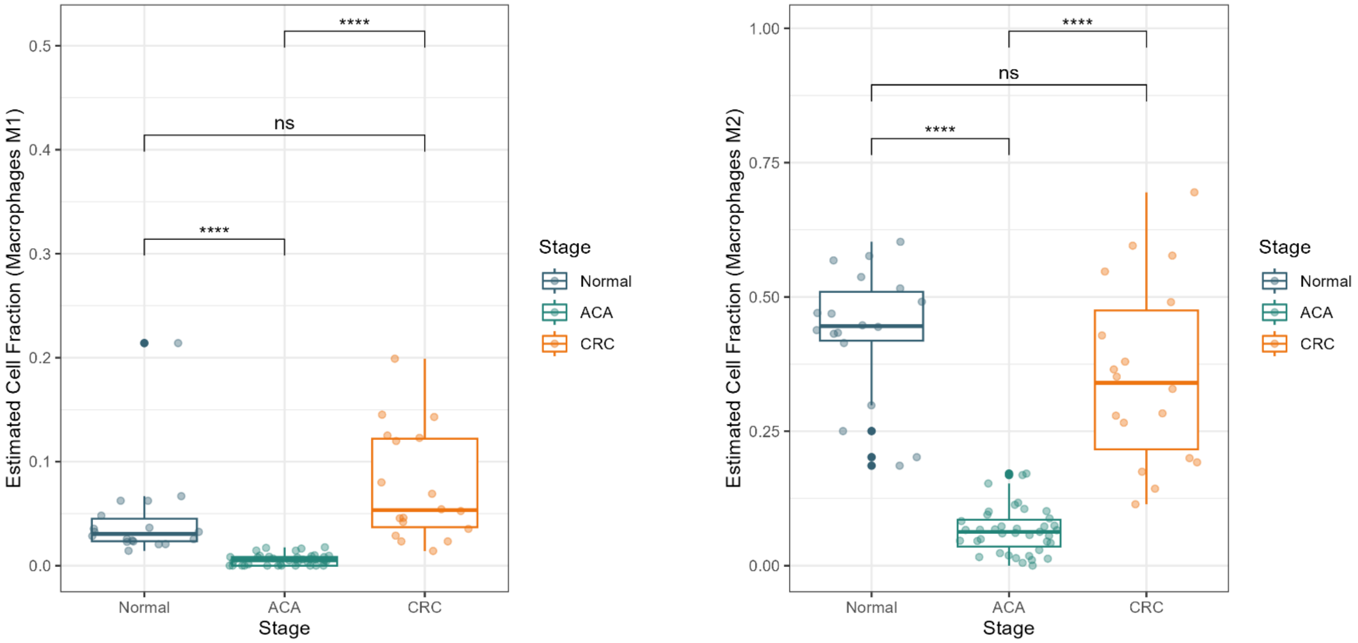

Supplementary Figure S5. Cell type fraction of M1 and M2 macrophages.

Box plots depicting the estimated cell fractions for M1 (left) and M2 (right) macrophages. The color of the graph indicate the stage of the sample.

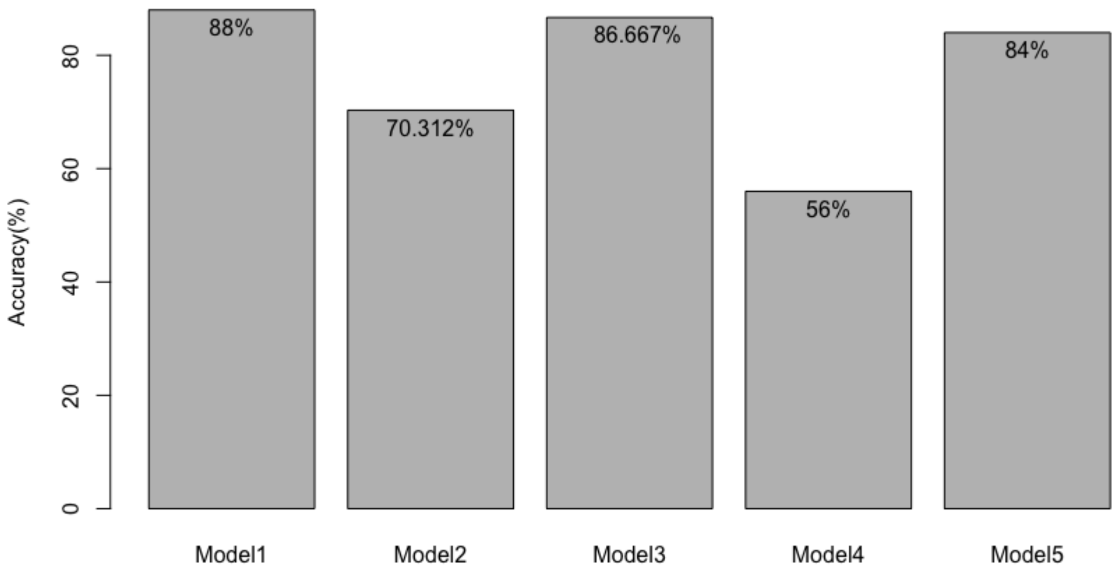

Supplementary Figure S6. The bar plots showing the accuracy of each multinomial logistic regression model. The accuracy values are annotated over each bar.

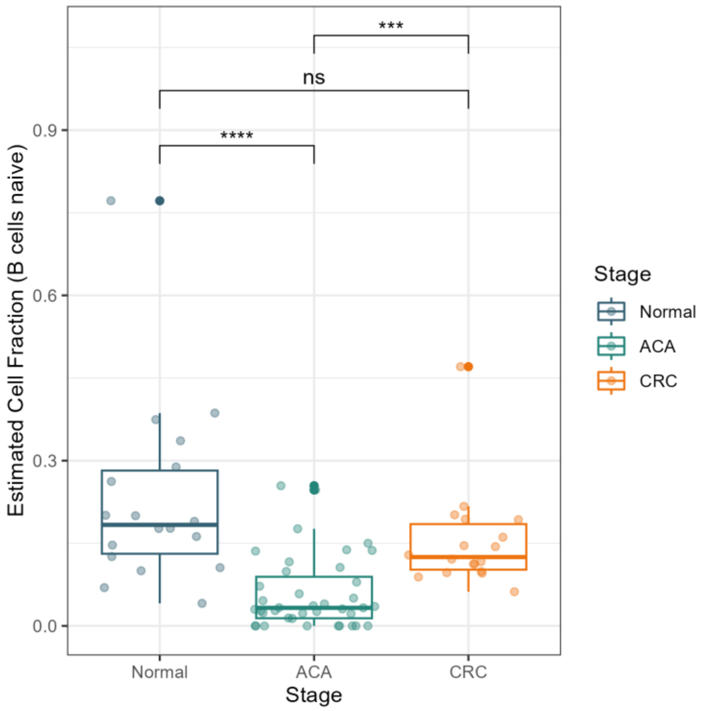

Supplementary Figure S7. Cell type fraction of naïve B-cells.

Box plots depicting the estimated cell fractions for naïve B-cells. The color of the graph indicate the stage of the sample.

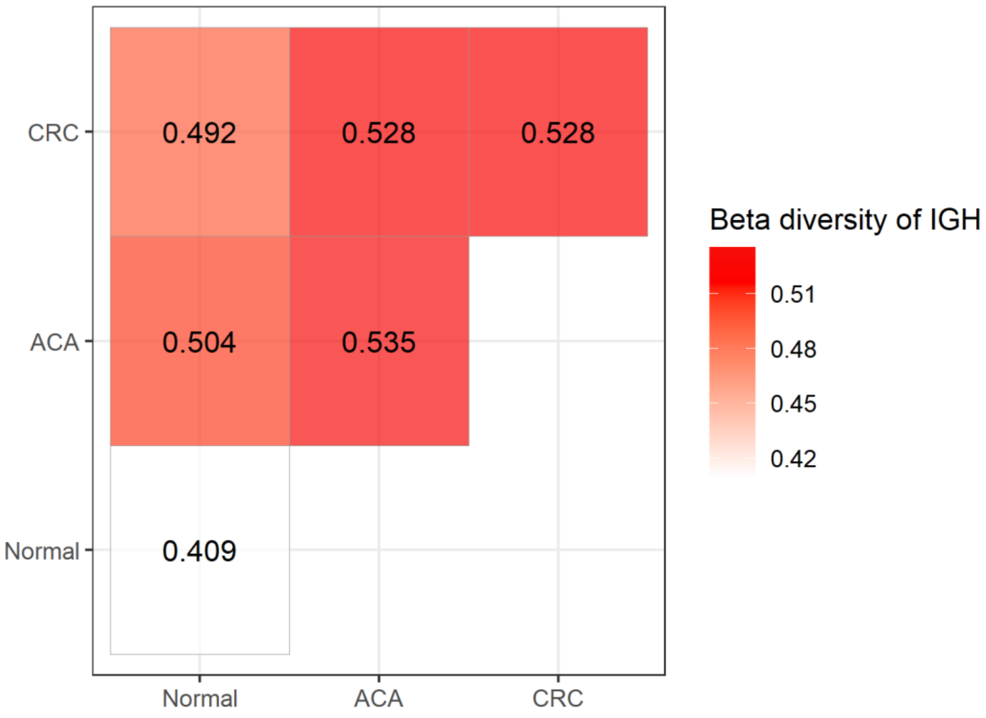

Supplementary Figure S8. Beta diversity analysis of IGH recombination patterns.

Heatmap showing the results of beta-diversity analysis for IgH recombination patterns between the sample pairs. Beta-diversity was calculated based on Sørensen-Dice index. The color corresponds to the mean beta diversity of IGH recombination patterns.

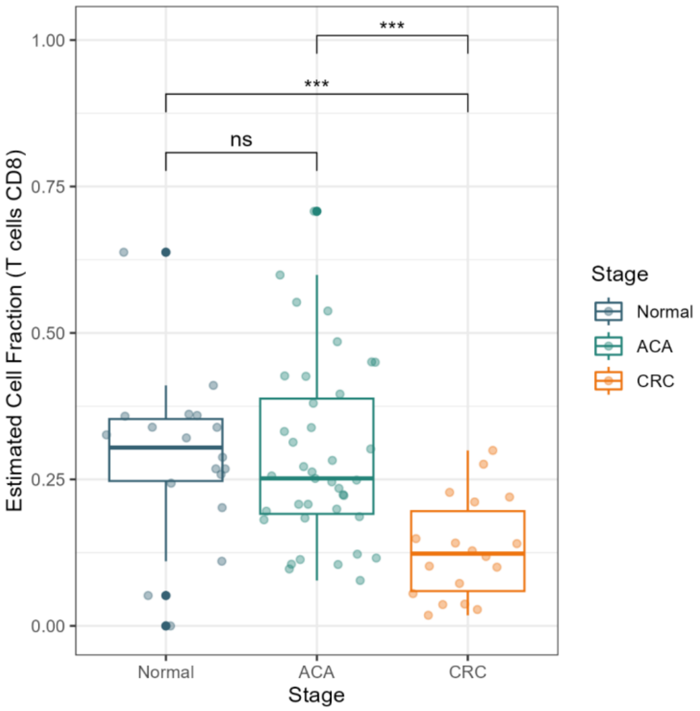

Supplementary Figure S9. Cell type fraction of CD8 T cells.

Box plots depicting the estimated cell fractions for CD8 T cells. The color of the graph indicate the stage of the sample.

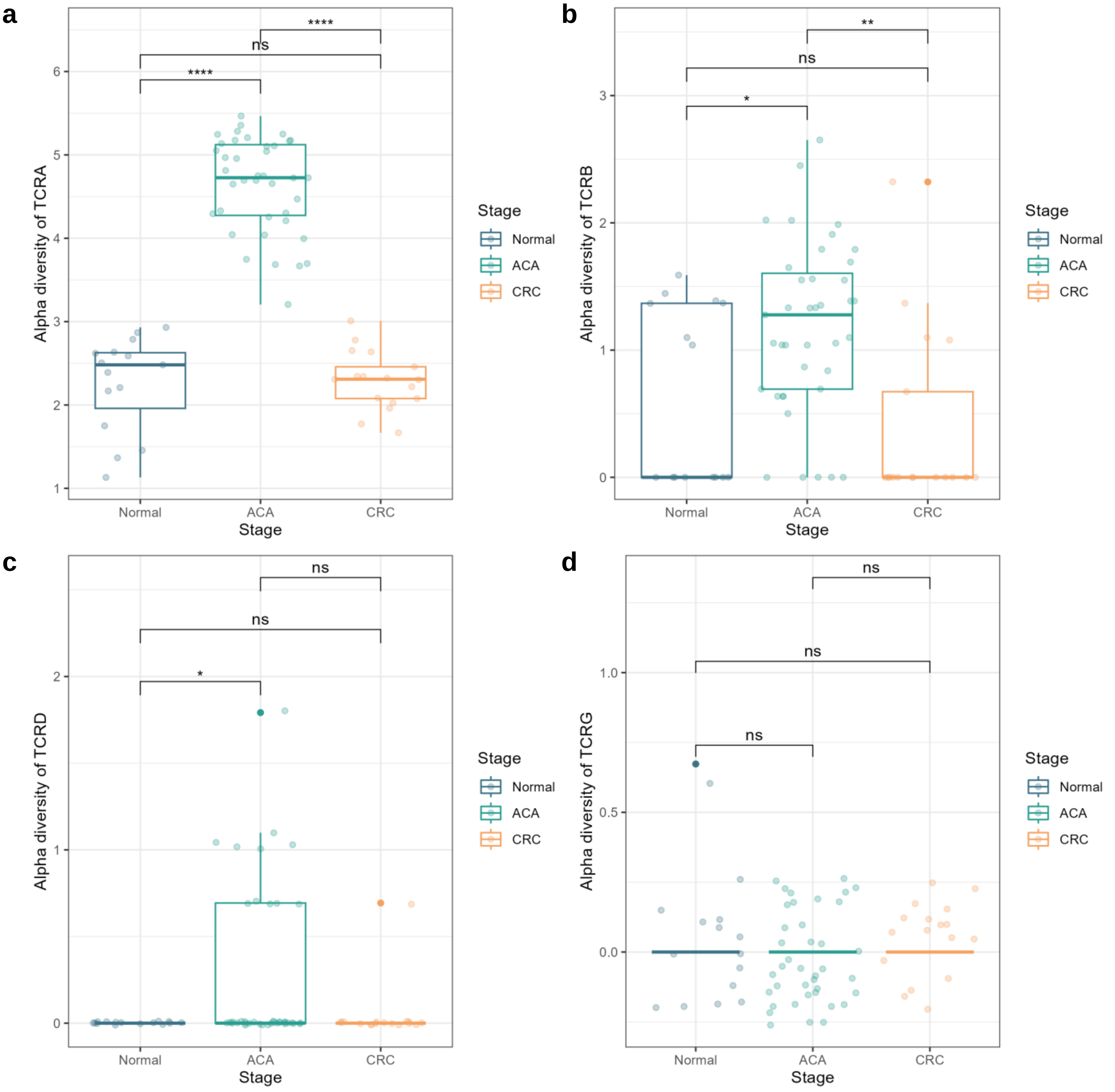

Supplementary Figure S10. TCR repertoire analysis across the CRC continuum.

The box plots of the immune repertoire diversity analysis: a) TCRA, b) TCRB, c) TCRD and d) TCRG chain. The color of the graph corresponds to the sample stages (normal: blue; ACA: green; CRC: yellow).

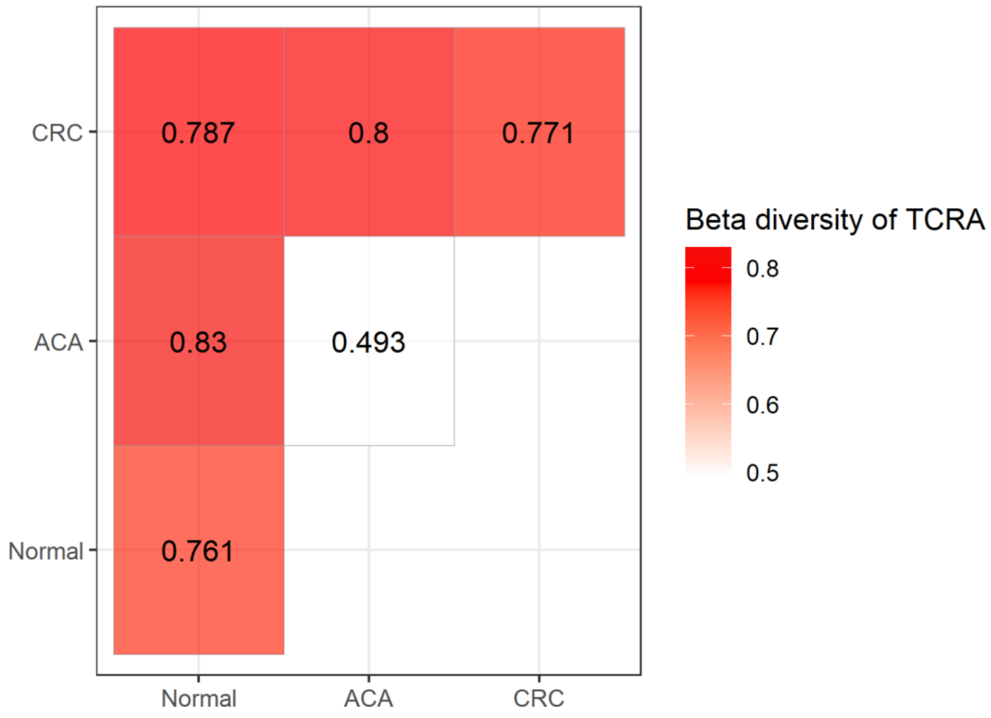

Supplementary Figure S11. Beta diversity analysis of TCRA recombination patterns.

Heatmap showing the results of beta-diversity analysis for TCRA recombination patterns between the sample pairs. Beta-diversity was calculated based on Sørensen-Dice index. The color corresponds to the mean beta diversity of TCRA recombination patterns.

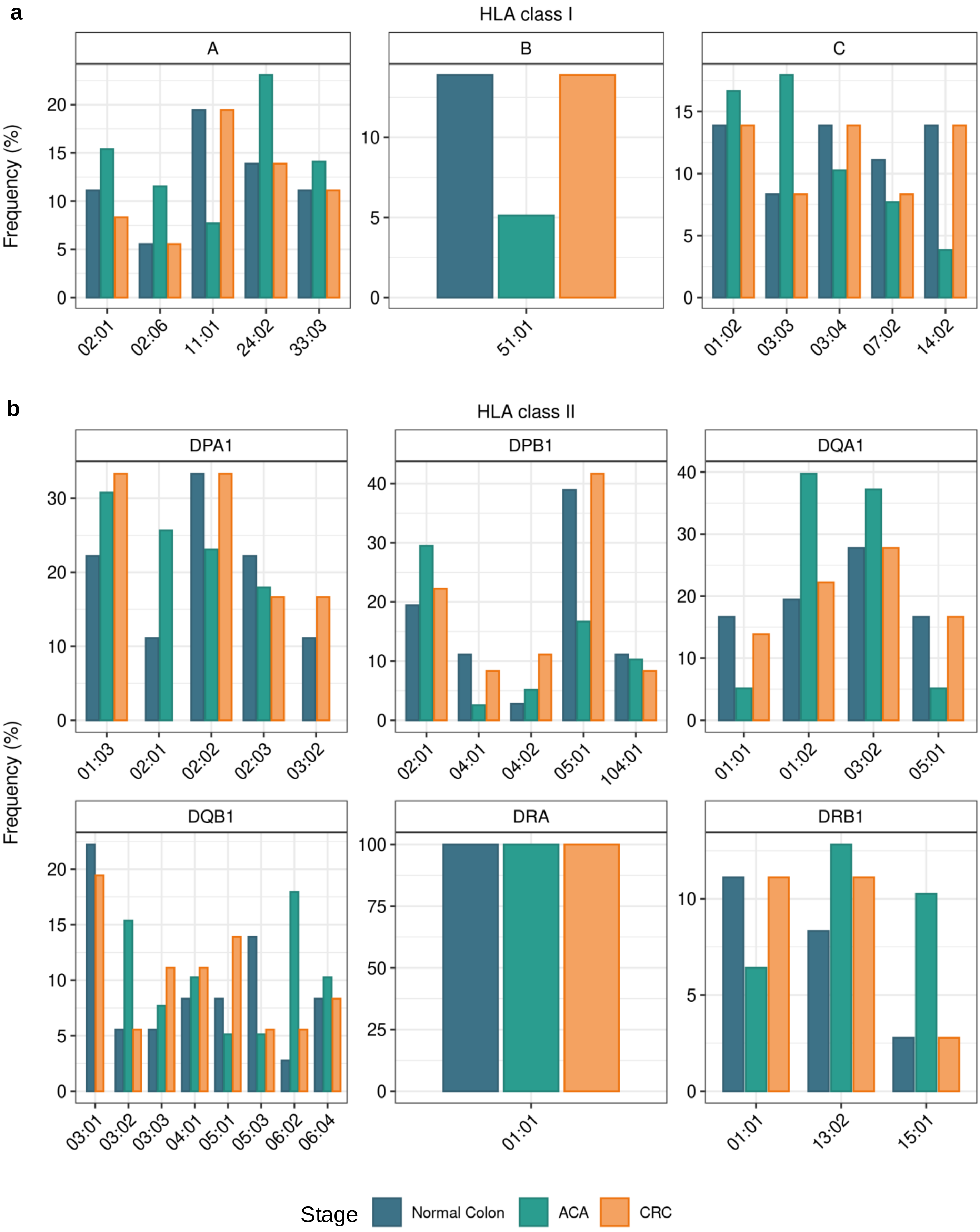

Supplementary Figure S12. HLA allele typing results using seq2HLA software.

Bar plot depicting the allele type frequencies for each class of HLA genes: a) Class I and b) Class II.
